# Venetoclax-ponatinib for T315I/compound-mutated Ph+ acute lymphoblastic leukemia

**DOI:** 10.1038/s41408-022-00621-9

**Published:** 2022-01-28

**Authors:** Huafeng Wang, Chang Yang, Ting Shi, Yi Zhang, Jiejing Qian, Yungui Wang, Yongxian Hu, Liping Mao, Xiujin Ye, Fang Liu, Zhenfang Xi, Lihong Shou, Caiyun Fu, Hua Naranmandura, Jie Jin, Hong-Hu Zhu

**Affiliations:** 1 Department of Hematology, The First Affiliated Hospital, Zhejiang University School of Medicine, Hangzhou, PR China; 2 Zhejiang Provincial Key Lab of Hematopoietic Malignancy, Zhejiang University, Hangzhou, Zhejiang PR China; 3 Institute of Hematology, Zhejiang University, Hangzhou, Zhejiang PR China; 4 Zhejiang University Cancer Center, Hangzhou, Zhejiang PR China; 5 Department of Public Health, Zhejiang University School of Medicine, Hangzhou, PR China; 6 Bone Marrow Transplantation Center, the First Affiliated Hospital, Zhejiang University School of Medicine, Hangzhou, PR China; 7 Department of Hematology, Chengdu Military General Hospital, Chengdu, Sichuan P.R. China; 8 Department of Hematology, Linfen People’s Hospital, Linfen, Shanxi PR China; 9 Department of Hematology, The Central Hospital of Huzhou City, Huzhou, People’s Republic of China; 10 College of Life Sciences and Medicine, Zhejiang Sci-Tech University, Hangzhou, China; 11 Zhejiang Laboratory for Systems & Precision Medicine, Zhejiang University Medical Center, Hangzhou, China

**Keywords:** Acute lymphocytic leukaemia, Clinical trials

The outcome of Philadelphia chromosome-positive acute lymphoblastic leukemia (Ph+ ALL) has greatly improved in the tyrosine kinase inhibitor (TKI) era and is moving to a chemo-free era using dasatinib and blinatumomab [1, 2]. However, the outcome of T315I/compound-mutated Ph+ ALL patients is dismal [3–5]. Our previous study, which included 38 Ph+ ALL patients harboring the T315I mutation, showed a poor outcome, although some patients received hematopoietic stem cell transplantation (HSCT) or chimeric antigen receptor (CAR) T cell therapy [5], which represents an unmet need for new therapeutic approaches for this population. Recently, bcl-2 inhibitors have shown promising therapeutic activity in Ph+ ALL in vitro [6–8]. Lenoard et al showed that the combination of TKIs and venetoclax has synergistic antileukemic efficacy in Ph+ ALL patient xenografted immunodeficient mice [7]. Moreover, Scherr et al. reported the curative potential of venetoclax-TKI-dexamethasone in a BCR-ABL + mouse model [8], thereby suggesting a novel treatment strategy for Ph+ ALL. Here, we report the promising clinical results of a series of relapsed Ph+ ALL patients harboring the T315I mutation treated with venetoclax, ponatinib, and dexamethasone (VPD).

We retrospectively analyzed relapsed/refractory (R/R) Ph+ ALL patients with T315I/compound-mutation who were treated with venetoclax (100 mg d1, 200 mg d2, 400 mg d3–28), ponatinib (45 mg d1–28) and dexamethasone (0.15 mg/kg d1–21, 0.075 mg/kg d22–28) (VPD regimen) between January 2020 and May 2021 (Fig. 1A). Informed consent was obtained from all the patients. The last follow-up date is May 20, 2021. Response assessments were performed according to the NCCN guidelines for acute lymphoblastic leukemia (version 2. 2021) [9]. Event-free survival (EFS) was defined as the time from complete remission (CR) after the VPD regimen until relapse, death, or last follow-up date, whichever occurred first. Overall survival (OS) was defined as the time from the start of the VPD regimen until death or the last follow-up date, whichever occurred first. Adverse events were graded according to National Cancer Institute Common Terminology Criteria for Adverse Events, version 5.0. Bcr/abl kinase domain mutation analysis was performed by using direct Sanger sequencing. All statistical analyses were conducted using Prism software version 7.0 (GraphPad Software, La Jolla, CA, USA).

**Fig. 1:**
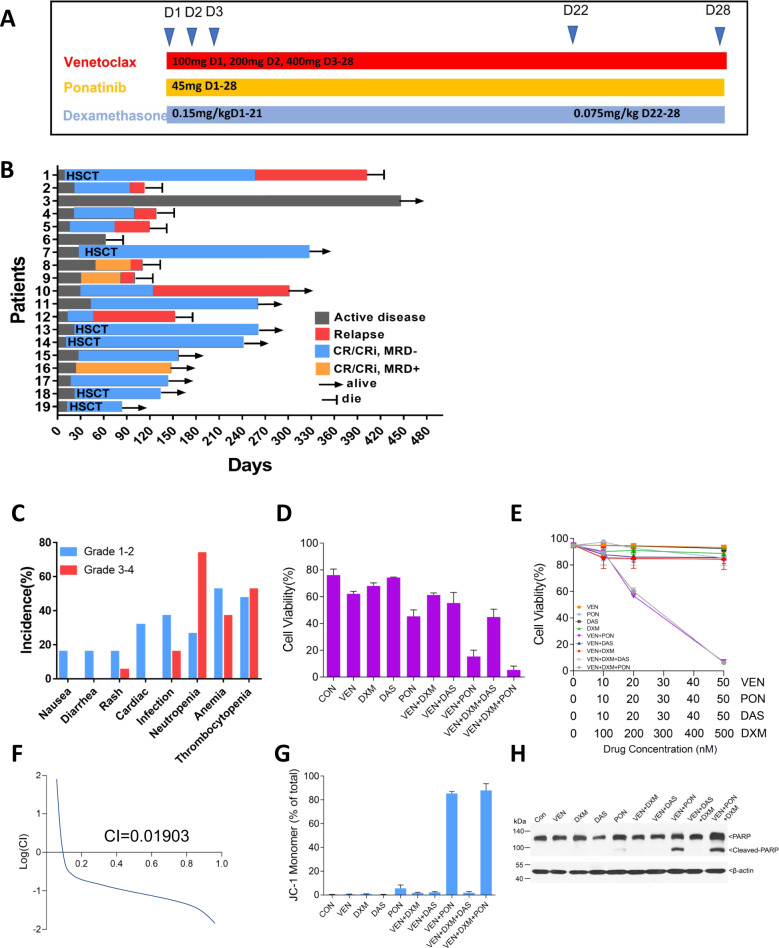
Clinical response and In vitro results of the VPD regimen. **A** Trial scheme. **B** Shown is the clinical outcome for the 19 refractory/relapsed Ph+ ALL patients with T315I or compound mutation who were treated with a VPD regimen. Response assessments were performed according to the NCCN guidelines Acute Lymphoblastic Leukemia (version 2. 2021). The indicated responses include a complete response (CR) and CR with incomplete count recovery (CRi). **C** Adverse Events of VPD regimen. **D** Mononuclear cells separated from one Ph+ ALL patient harboring T315I mutation were treated with venetoclax (VEN), ponatinib (PON), dasatinib (DAS), dexamethasone (DXM) alone or in combination at the indicated concentrations for 24 h. Apoptosis, as indicated by flow cytometry after Annexin V/PI double staining. **E**–**H** BaF3 cells expressing p190 BCR-ABL T315I mutation were treated with VEN, PON, DAS, DXM alone or in combination at the indicated concentrations for 24 h. Apoptosis, as indicated by flow cytometry after Annexin V/PI double staining (**E**), combination index (CI) of three drugs by using CompuSyn software (**F**), mitochondrial were stained with JC-1 (**G**), and western-blot analysis of PARP and cleaved-PARP expression (**H**). Notes: MRD minimal residual disease, as assessed by flow cytometry. HSCT hematopoietic stem-cell transplantation.

19R/R Ph+ ALL patients with the T315I/compound mutation received the VPD regimen, which included 12 males and 7 females, with a median age of 42 years (range 22 years to 74 years). Of these patients, 17 patients had de novo Ph+ ALL, 15 had BCR/ABL p190, and 2 had BCR/ABL p210. Two patients had secondary ALL accelerated from CML, with BCR/ABL p210. All patients had the T315I mutation alone or compound mutation when relapsed before VPD regimen treatment, including 15 patients with the T315I mutation alone and 4 with compound kinase mutations (100% T315I + 50% E255K/V, 100% T315I + 100% E279K, 100% T315I + 100% Y253H, and 54% G250E + 36% F359V for each). BCR-ABL1 compound mutants were defined as harboring ≥2 mutations in the same BCR-ABL1 allele [10]. The first three patients were considered as compound mutation because of their individual 100% T315I mutant although we just performed Sanger sequencing but not clonal sequencing. While the patient with 54% G250E and 36% F359V mutant was not verified by clonal sequencing because of running out of sample.19 patients had previously received a median of 3 lines of salvage therapy (range: 1–6), including all patients who failed TKI (imatinib, dasatinib, nilotinib, flumatinib, or ponatinib) with or without chemotherapy, 5 patients who failed CAR T cell therapy, 1 who failed venetoclax, and 1 who failed allogeneic hematopoietic stem cell transplantation (allo-HSCT) (Table 1, Table S1).

**Table 1:** Baseline characteristics of Ph+ ALL patients.

Characteristics		All patients (*n* = 19)
*Age, years*		
Median	42	
Range	22–74	
*Sex, n (%)*		
Male	12 (63.2)	
Female	7 (36.8)	
*BCR/ABL, n (%)*		
P190	15 (78.9)	
P210	4 (21.1)	
*ABL kinase domain, n (%)*		
T315I alone	15 (78.9)	
T315I and E255K/V	1 (5.3)	
T315I and E279K	1 (5.3)	
T315I and Y253H	1 (5.3)	
G250E and F359V	1 (5.3)	
*Previous lines of therapy, median(range)*	3 (1–6)	
Failure to TKI ± chemotherapy, *n* (%)	19 (100)	
Failure to venetoclax, *n* (%)	1 (5.3)	
Failure to CAR-T therapy, *n* (%)	5 (26.3)	
Failure to allo-HSCT, *n* (%)	1 (5.3)	
*Response after one cycle of VPD, n (%)*		
CR	13 (68.4)	
CRi	4 (21.1)	
NR	2 (10.5)	
*MRD-FCM in responder, n (%)*		
Negative	14 (82.4)	
Positive	3 (17.6)	
MMR in responder, *n* (%)	11 (64.7)	
CMR in responder, *n* (%)	8 (47.1)	
TOR in responder, median (range)	25 (10–53)	
*Post-remission, n (%)*		
Allo-HSCT group	6 (35.3)	
VPD group	11 (64.7)	
Relapse, *n* (%)	8 (47.1)	
Allo-HSCT group	1 (16.7)	
VPD group	7 (63.6)	

*TKIs* tyrosine kinase inhibitors, *CAR-T* chimeric antigen receptor-T, *Allo-HSCT* allogeneic hematopoietic cell transplantation, *CR* complete remission, *CRi* CR with incomplete count recovery, *NR* no remission, *MRD-FCM* minimal residual disease detected by flow cytometry, *TOR* time to response, *TOMMR* time to major molecular remission. *From the starting day of the VPD regimen.

After one cycle, 17 patients (89.5%) achieved CR/CRi [14 minimal residual diseases (MRD)—negative by flow cytometry; 11 major molecular remission (MMR); 8 complete molecular remissions (CMR)] (Fig. 1B, Table 1, Table S1). For post-remission treatment, 6 patients who attained CMR directly received allo-HSCT, and the remaining 11 patients continued to receive the VPD regimen. Four of 11 patients attained CMR later during VPD consolidation treatment. The other two patients who did not respond did not achieve CR/CRi later. For 6 patients who received allo-HSCT, the type of donor, intensity of conditioning regimen, and timing of BMT are listed in Table S2. Five patients received haploidentical transplantation, and the other patient received 9/10 unrelated donor transplantation. All 6 patients received a modified busulfan (BU) and cyclophosphamide (CY) conditioning regimen prior to allo-HSCT when they attained CMR after one cycle of the VPD regimen. Subsequently, relapse occurred in 1/6 [allo-HSCT group)] and 7/11 (VPD consolidation group). Of the 6 patients who received allo-HSCT, 2/6 (patient 1 and patient 19) received 45 mg of ponatinib per day as maintenance treatment, and 4 other patients did not receive maintenance treatment. At a median follow-up of 259 days, the median EFS and OS of patients starting VPD treatment were 242 and 400 days, respectively.

Adverse events of the VPD regimen were listed in Fig. 1C. Grade 3–4 neutropenia, anemia, and thrombocytopenia occurred in 73.7%, 36.8%, and 52.6% of patients, respectively. A total of 5.3% and 16% of patients had grade 3–4 rash and infection, respectively. Patient 12 with grade 3 rash and patient 15 with grade 2 rash reduced the ponatinib dose from 45 mg per day to 30 mg per day. No tumor lysis syndrome or death occurred. 7/19 patients were treated safely outpatient.

Moreover, venetoclax had a strong synergistic effect with ponatinib and dexamethasone on inducing apoptosis of primary blast cells and BaF3 cells expressing p190 BCR/ABL with the T315I mutation in vitro, with a combination index of 0.019 when the suppression rate was 0.05, while the effect was significantly decreased when ponatinib was replaced by dasatinib (Fig. 1D–F). A prominent change in mitochondrial membrane potential and cleavage of PARP was also observed in the triple-combination treatment group (Fig. 1G, H). Unfortunately, only one patient sample was available, and more patient samples need to be verified in the future.

For T315I/compound-mutated Ph+ ALL, the VPD regimen exhibited an 89.5% CR/CRi rate, with deep molecular remission (57.9% MMR), while ponatinib alone showed a 41% hematologic response [3], which was supported by preclinical data suggesting that TKIs and venetoclax are highly synergistic in BCR-ABL + cells in vitro. A total of 7/11 and 1/6 patients subsequently relapsed in the continuous VPD and allo-HSCT post-remission treatment groups, respectively, suggesting that bridging to allo-HSCT after remission is warranted. Moreover, novel compounds such as blinatumomab showed a preliminary efficacy [11]. Comparisons of efficacy in different salvage regimens for R/R Ph+ ALL with T315I or compound mutations are listed in Table S3. In summary, the VPD regimen provides a novel treatment for T315I/compound-mutated R/R Ph+ ALL under a complete oral and chemo-free model. A clinical trial also using a similar VPD regimen for the treatment of R/R Ph+ ALL is ongoing now, and the results of a phase 1 portion of this study including nine patients also showed that the combination of ponatinib, venetoclax, and dexamethasone in R/R Ph+ ALL is safe and effective [12].

## Supplementary information

Supplemental Material
