# Supplementary material for: Venetoclax-ponatinib for T315I/compound-mutated Ph+ acute lymphoblastic leukemia
Source: Blood Cancer J. 2022 Jan 28;12(1):20. doi: 10.1038/s41408-022-00621-9 (PMC8799711; doi:10.1038/s41408-022-00621-9)
Supplement: Supplementary file 1 — Supplemental Material [file 41408_2022_621_MOESM1_ESM.docx]

**Supplemental Materials**

**Table S1 Characteristic of Ph+ ALL patients with VPD regimen**

| Patient No. | Age | Sex | BCR/ABL | ABL kinase domain mutation | Previous lines of Therapy(no.) | Failure to TKI +chemo, venetoclax, CAR-T cell therapy, allo-HSCT | Response | MRD-FCM | BCR/ABL | TOR(day) | TOMMR(day) | Post-remission | Post-HSCT maintenance/TKI | Relapse | EFS(day)* | OS（day）* | Survival status |
| --- | --- | --- | --- | --- | --- | --- | --- | --- | --- | --- | --- | --- | --- | --- | --- | --- | --- |
| 1 | 38 | M | P190 | T315I | 4 | Dasatinib + chemo, CAR-T(CD19,CD22) | CR | <0.01% | 0 | 10 | 10 | Allo-HSCT | Ponatinb | Yes | 242 | 400 | Die |
| 2 | 26 | M | P190 | T315I | 6 | Dasatinib + chemo, CAR-T(CD19), venetoclax | CR | <0.01% | 0.0091% | 25 | 25 | VPD | / | Yes | 75 | 111 | Die |
| 3 | 74 | M | P190 | T315I | 2 | Dasatinib or Ponatinib + chemo | NR | 34.65% | 29% | / | / | / | / | / | / | 445+ | Alive |
| 4 | 58 | M | P190 | T315I | 2 | Imatinib + chemo | CR | <0.01% | 0.0033% | 25 | 25 | VPD | / | Yes | 79 | 127 | Die |
| 5 | 56 | F | P190 | T315I | 3 | Imatinib or Dasatinib + chemo | CRi | <0.01% | 0.0018% | 18 | 26 | VPD | / | Yes | 53 | 118 | Die |
| 6 | 48 | F | P190 | T315I | 2 | Imatinib or Dasatinib + chemo | NR | 14.325% | 65% | / | / | / | / | / | / | 61 | Die |
| 7 | 28 | M | P210 | G250E, F359V | 4 | Imatinib or Dasatinib or Ponatinib+chemo;CAR-T(CD19) | CR | <0.01% | 0 | 31 | 31 | Allo-HSCT | No | No | 291+ | 322+ | Alive |
| 8 | 54 | F | P210 | T315I | 3 | Imatinib or Nilotinib or Flumatinib + chemo | CRi | 3.06% | 47% | 53 | / | VPD | / | Yes | 37 | 109 | Die |
|  |  |  |  |  |  |  |  |  |  |  |  |  |  |  |  |  |  |
| 9 | 66 | F | P190 | T315I E255K/V, | 6 | Imatinib or Ponatinib + chemo | CRi | 1.95% | 13.20% | 31 | / | VPD | / | Yes | 49 | 100 | Die |
| 10 | 61 | F | P190 | T315I | 5 | Dasatinib or Imatinib + chemo | CR | <0.01% | 0 | 30 | 30 | VPD | / | Yes | 90 | 300+ | Alive |
| 11 | 36 | M | P210 | T315I | 2 | Imatinib or Dasatinib+ chemo | CR | <0.01% | 1.32% | 45 | / | VPD | / | No | 214+ | 259+ | Alive |
| 12 | 63 | F | P190 | T315I, E279K | 3 | Dasatinib+ chemo  CAR-T(CD19,CD22) | CR | <0.01% | 15% | 16 | / | VPD | / | Yes | 30 | 151 | Die |
| 13 | 24 | F | P190 | T315I | 4 | Dasatinib+ chemo  CAR-T（CD19） | CR | <0.01% | 0 | 25 | 25 | Allo-HSCT | No | No | 234 | 259 | Alive |
| 14 | 22 | M | P190 | T315I | 1 | Dasatinib+ chemo | CR | <0.01% | 0 | 14 | 14 | Allo-HSCT | No | No | 216+ | 240+ | Alive |
| 15 | 42 | M | P210 | T315I | 4 | Dasatinib or Ponatinib+chemo | CR | <0.01% | 22.63% | 29 | / | VPD | / | No | 127+ | 156+ | Alive |
| 16 | 38 | M | P190 | T315I,  Y253H | 4 | Imatinib or Dasatinib+ chemo, allo-HSCT | CRi | 1.27% | 0.556% | 27 | / | VPD | / | No | 119+ | 146+ | Alive |
| 17 | 52 | M | P190 | T315I | 2 | Flumatinib+chemo | CR | <0.01% | 0 | 23 | 23 | VPD | / | No | 119+ | 142+ | Alive |
| 18 | 35 | M | P190 | T315I | 2 | Imatinib or Dasatinib + chemo | CR | <0.01% | 0 | 25 | 25 | Allo-HSCT | No | No | 107+ | 142+ | Alive |
| 19 | 36 | M | P190 | T315I | 1 | Dasatinib+chemo | CR | <0.01% | 0 | 14 | 14 | Allo-HSCT | Ponatinib | No | 68+ | 82+ | Alive |

TKIs: tyrosine kinase inhibitors; CAR-T: Chimeric Antigen Receptor-T; chemo: chemotherapy; Allo-HSCT: Allogeneic hematopoietic cell transplantation; CR: Complete remission; CRi: CR with incomplete count recovery; NR：No remission; MRD-FCM: Minimal residual disease detected by flow cytometry; TOR: Time to response; TOMMR: Time to major molecular remission; EFS: event-free survival; OS: overall survival.

*From the starting day of VPD regimen

**Table S2: Allo-HSCT information of patients.**

| Patient No. | Type of donor | Intensity of conditioning regimen | | Timing of BMT | BCR/ABL |
| --- | --- | --- | --- | --- | --- |
| 1 | Haploididentical | | Modified BU/CY | After one cycle of VDP regimen | CMR |
| 7 | Haploididentical | | Modified BU/CY | After one cycle of VDP regimen | CMR |
| 13 | Haploididentical | | Modified BU/CY | After one cycle of VDP regimen | CMR |
| 14 | Haploididentical | | Modified BU/CY | After one cycle of VDP regimen | CMR |
| 18 | 9/10 unrelated donor | | Modified BU/CY | After one cycle of VDP regimen | CMR |
| 19 | Haploididentical | | Modified BU/CY | After one cycle of VDP regimen | CMR |

BMT：Bone marrow transplantation; BU: Busulfan; CY: Cyclophosphamide; CMR: Complete molecular remission.

**Table S3. Companion of different salvage regimens in prior heavy treated Ph+ ALL patients with T315I or compound mutation.**

| Regimen | Year | Prior heavy treated patients with T315I or compound mutation (n) | Salvage cycles (n.) | Prior alloHSCT | Prior CAR-T | Response | | | | |
| --- | --- | --- | --- | --- | --- | --- | --- | --- | --- | --- |
|  |  |  |  |  |  | CR/CRi/CRh | MRD-FCM in CR/CRi patients | MMR in CR/CRi patients | Relapse in CR/CRi patients without alloHSCT after rescue | Relapse in patients with alloHSCT after rescue |
| VPD regimen (Present study) | 2021 | 16 | 1 | 1 | 4 | 14/16 | 8/14 | 8/14 | 7/10 | 1/4 |
| VPD* regimen([1](#_ENREF_1)) | 2021 | 4 | NA | NA | NA | NA | NA | NA | NA | NA |
| Blinatumomab+ponatinib([2](#_ENREF_2)) | 2021 | 5  （1 F371L+Y253H；4 T315I） | 1-5 | 2 | 0 | 5/5 | NA | 5/5 | 4/4 | 0/1 |
| Blinatumomab+ponatinib([3](#_ENREF_3)) | 2017 | 1 | 2(1-3) | 0 | 0 | 0/1 | 0/1 | 0/1 | 0/1 | 0/0 |
| Blinatumomab([4](#_ENREF_4)) | 2017 | 10 (included relapse after first line treatment) | 1-2 | NA | 0 | 4/10 | 4/10 | 4/10 | NA | NA |
| Ponatinib([5](#_ENREF_5)) | 2013 | 22(included relapse after first line treatment, 7 compound mutation, 15 T315I alone) | 1-6 | NA | 0 | 8/22 | NA | 9/22(MCyR) | NA | NA |

Prior heavy treated: A second line or more therapies were used before; CAR-T: Chimeric Antigen Receptor-T; CR: Complete remission CRi: CR with incomplete count recovery; CRh: CR with partial hematological recovery; MRD-FCM: Minimal residual disease detected by flow cytometry; MMR: Major molecular remission; alloHSCT: Allogeneic hematopoietic cell transplantation; VPD: venetoclax 100mg d1, 200mg d2, 400mg d3-28, ponatinib 45mg d1-28 and dexamethasone 0.15mg/kg d1-21,0.075mg/kg d22-28; VPD* : venetoclax 20mg d1, 50mg d2,100mg d3, 200mg d4, 400mg d5-28 or 400mg d5,800mg d6-28, ponatinib 45mg d1-28 and dexamethasone 40mg d1-4 MCyR: Major cytogenetic remission.

**Reference**

1. Short NJ, Konopleva M, Kadia T, Kebriaei P, Daver N, Huang X, *et al.* An effective chemotherapy-free regimen of ponatinib plus venetoclax for relapsed/refractory Philadelphia chromosome-positive acute lymphoblastic leukemia. *Am J Hematol* **96**, E229-E232 (2021).

2. Couturier MA, Thomas X, Raffoux E, Huguet F, Berthon C, Simand C, et *al.* Blinatumomab + ponatinib for relapsed/refractory Philadelphia chromosome-positive acute lymphoblastic leukemia in adults. *Leuk Lymphoma* **62**, 620-629 (2021).

3. Assi R, Kantarjian H, Short NJ, Daver N, Takahashi K, Garcia-Manero G, et *al.* Safety and Efficacy of Blinatumomab in Combination With a Tyrosine Kinase Inhibitor for the Treatment of Relapsed Philadelphia Chromosome-positive Leukemia. *Cl lymph myelom leuk* **17**, 897-901(2017).

4. Martinelli G, Boissel N, Chevallier P, Ottmann O, Gökbuget N, Topp MS, et *al.* Complete Hematologic and Molecular Response in Adult Patients With Relapsed/Refractory Philadelphia Chromosome-Positive B-Precursor Acute Lymphoblastic Leukemia Following Treatment With Blinatumomab: Results From a Phase II, Single-Arm, Multicenter Study. *J Clin Oncol* **35**,1795-802(2017).

5. Cortes JE, Kim DW, Pinilla-Ibarz J, le Coutre P, Paquette R, Chuah C, *et al.* A phase 2 trial of ponatinib in Philadelphia chromosome-positive leukemias. *N Engl J Med* **369**, 1783-1796 (2013).
